# Structural basis of Lewis^b^ antigen binding by the *Helicobacter pylori* adhesin BabA

**DOI:** 10.1126/sciadv.1500315

**Published:** 2015-08-14

**Authors:** Naim Hage, Tina Howard, Chris Phillips, Claire Brassington, Ross Overman, Judit Debreczeni, Paul Gellert, Snow Stolnik, G. Sebastiaan Winkler, Franco H. Falcone

**Affiliations:** 1 School of Pharmacy, University of Nottingham, University Park, Nottingham NG7 2RD, UK.; 2 Discovery Sciences, Innovative Medicines and Early Development, AstraZeneca R&D, Alderley Park, Cheshire SK10 4TG, UK.; 3 Discovery Sciences, Innovative Medicines and Early Development, AstraZeneca R&D, Darwin Building, 310 Cambridge Science Park, Milton Road, Cambridge CB4 0WG, UK.; 4 Pharmaceutical Development, AstraZeneca R&D, Charter Way, Macclesfield, Cheshire SK10 2NA, UK.

**Keywords:** Helicobacter pylori, adhesin, LewisB, BabA, X-ray crystallography, bacterial adhesion

## Abstract

*Helicobacter pylori* is a leading cause of peptic ulceration and gastric cancer worldwide. To achieve colonization of the stomach, this Gram-negative bacterium adheres to Lewis^b^ (Le^b^) antigens in the gastric mucosa using its outer membrane protein BabA. Structural information for BabA has been elusive, and thus, its molecular mechanism for recognizing Le^b^ antigens remains unknown. We present the crystal structure of the extracellular domain of BabA, from *H. pylori* strain J99, in the absence and presence of Le^b^ at 2.0- and 2.1-Å resolutions, respectively. BabA is a predominantly α-helical molecule with a markedly kinked tertiary structure containing a single, shallow Le^b^ binding site at its tip within a β-strand motif. No conformational change occurs in BabA upon binding of Le^b^, which is characterized by low affinity under acidic [*K*_D_ (dissociation constant) of ~227 μM] and neutral (*K*_D_ of ~252 μM) conditions. Binding is mediated by a network of hydrogen bonds between Le^b^ Fuc1, GlcNAc3, Fuc4, and Gal5 residues and a total of eight BabA amino acids (C189, G191, N194, N206, D233, S234, S244, and T246) through both carbonyl backbone and side-chain interactions. The structural model was validated through the generation of two BabA variants containing N206A and combined D233A/S244A substitutions, which result in a reduction and complete loss of binding affinity to Le^b^, respectively. Knowledge of the molecular basis of Le^b^ recognition by BabA provides a platform for the development of therapeutics targeted at inhibiting *H. pylori* adherence to the gastric mucosa.

## Teaser

X-ray structure of BabA bound to Lewis^b^ reveals molecular interactions used by *H. pylori* to colonize the gastric mucosa.

## INTRODUCTION

*Helicobacter pylori* is one of the most common causative agents of bacterial infections in the world with more than one-half of the global population affected (*1*). This microaerophilic, Gram-negative bacterium colonizes the human gastric mucosa and is a strong risk factor for the development of peptic ulceration, gastric adenocarcinoma, and gastric mucosa–associated lymphoid tissue (MALT) lymphoma. Infection by *H. pylori* can persist in the harsh conditions of the stomach for decades (*2*, *3*). This is achieved through unique evolutionary adaptations, including the use of a variety of outer membrane proteins to adhere to glycan moieties on the gastric epithelium (*4*, *5*). Through this mechanism, clearance from the stomach during mucus turnover is evaded, and local colonization is sustained (*6*).

The blood group antigen–binding adhesin (BabA) is one of the best-characterized adhesion proteins of the bacterium. It contains two domains: an N-terminal extracellular host-binding domain (*7*–*9*) and a C-terminal outer membrane–spanning domain predicted to form a β-barrel structure similar to that of known porins (*10*). Most disease-causing strains express this adhesin, and its presence on the bacterium correlates with enhanced colonization and virulence (*11*–*14*). BabA mediates *H. pylori* attachment by binding to fucosylated histo-blood group antigens found on gastric epithelial cells and mucin (fig. S1). The most studied of these interactions is between BabA and the Lewis^b^ (Le^b^) antigen. This difucosylated oligosaccharide is abundantly expressed by the healthy gastric mucosa of most individuals in the Western population with the O phenotype, who are also the most susceptible to peptic ulcer disease (*15*, *16*). However, the BabA binding site responsible for Le^b^ attachment has remained elusive.

The lack of structural information for BabA hinders the development of strategies to treat antibiotic-resistant infections through the inhibition of *H. pylori* attachment to the gastric mucosa. The potential of this approach has already been demonstrated for uropathogenic *E. coli*, where inhibition of its FimH adhesin can successfully treat and prevent infection (*17*, *18*). Here, we investigated the molecular interactions that mediate BabA binding to Le^b^ antigens by x-ray crystallography. We present the crystal structure of the extracellular domain of BabA in the absence and presence of Le^b^ at 2.0- and 2.1-Å resolutions, respectively.

## RESULTS

### Crystal structure of BabA

**Fig. 1:**
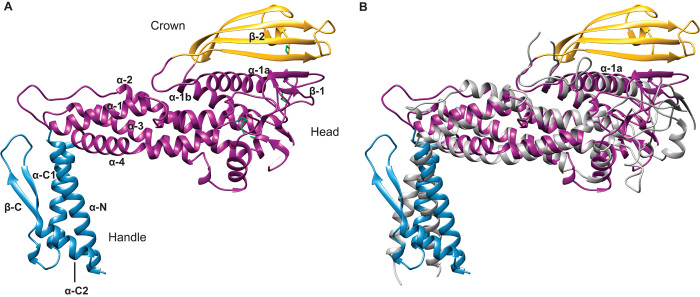
Comparison of the BabA and SabA extracellular domain crystal structures. (**A**) Crystal structure of the BabA extracellular domain. Indicated are the handle (blue) and head regions (dark magenta) and the crown β-strand unit (gold). The four disulfide bridges are represented as green sticks. (**B**) Superimposition of the extracellular domains of BabA and SabA (gray).

We first determined the crystal structure of the N-terminal extracellular domain of BabA, from strain J99 (*19*), to 2.0-Å resolution (table S1). BabA contains two predominantly α-helical regions—a handle and head region, and a third β-sheet motif located on top of the head region. We term this β-strand unit the crown (Fig. 1A).

The handle region, containing both the N and C termini of the extracellular domain, forms an α+β unit. The N-terminal helix (α-N) forms a two-helix antiparallel coiled-coil bundle with a C-terminal helix (α-C1) of similar approximate length. This C-terminal helix is followed by a two-strand antiparallel β-sheet (β-C) before ending with a short α-helix (α-C2), which packs against α-N in an antiparallel orientation. In the native BabA protein, the highly conserved putative β-barrel transmembrane domain succeeds the α-C2 helix (fig. S2). The core of the head region is composed of a four-helix antiparallel coiled-coil bundle, similar to a tetratricopeptide repeat motif (α-1 to α-4), at a near perpendicular angle to the handle region creating the markedly kinked tertiary structure. The connecting features between these four helices are (i) a loop containing a short α-helix (followed by a disordered region of seven amino acids) between α-3 and α-4; (ii) a 20–amino acid loop between α-2 and α-3; and (iii) a ~200–amino acid segment (including a disordered region of 8 amino acids) between the α-1 and α-2 helices. This connecting segment, which extends out of the core of the head region, contains a small β-sheet (β-1), a pair of interacting α-helices (α-1a and α-1b), and the crown—a four-strand antiparallel β-sheet at the highest tip of the protein (β-2).

BabA belongs to the *Helicobacter* outer membrane porins (Hop) family, from which the crystal structure of the functionally related sialic acid–binding adhesin (SabA), from *H. pylori* strain 26695, has been determined (*20*). The extracellular domain of SabA 26695, which shares only 26% amino acid sequence identity with that of BabA J99 (fig. S3), was identified as the single most related structure in the Protein Data Bank (PDB) database [Fig. 1B; root mean square deviation (RMSD) = 3.7 Å for all Cα atoms (*21*)]. Superimposition of BabA and SabA shows that the α-1a helix is a common characteristic. This feature has been suggested to form part of the glycan binding cavity of SabA, which recognizes sialyl-Lewis^x^ (SLe^x^) antigens found on cancerous and inflamed gastric tissue (*20*). Whereas both proteins share highly similar three-dimensional folds, the four-strand antiparallel β-sheet crown of BabA is altogether absent in SabA.

No DNA or protein sequences with similarity to the crown in *H. pylori* or any other organism were identified in NCBI (National Center for Biotechnology Information) nucleotide sequence and nonredundant protein sequence databases.

### Crystal structure of BabA in complex with Le^b^

**Fig. 2:**
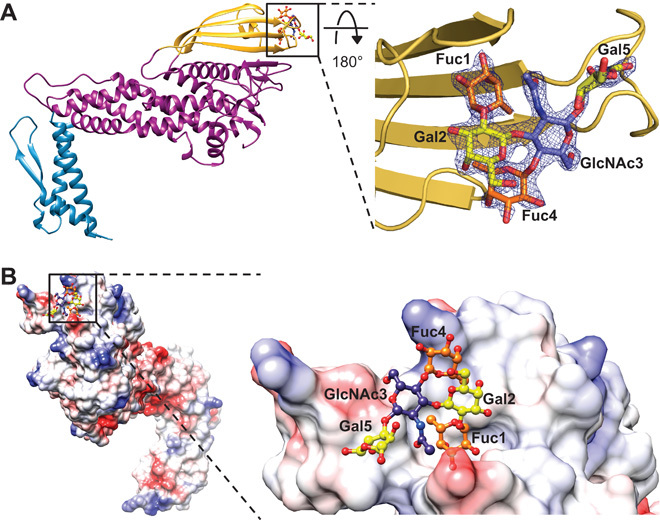
The Le^b^ binding site of BabA. (**A**) Le^b^ binds to BabA at one edge of the crown. The electron density map around Le^b^ (2*F*_o_ − *F*_c_ map, contoured at 2.0σ) in complex with BabA is shown. (**B**) An electrostatic surface representation shows Le^b^ binding to a shallow, solvent-exposed pocket at the tip of BabA containing charged and neutral patches. Fucose, galactose, and *N*-acetylglucosamine residues are colored orange, yellow, and blue, respectively.

To obtain structural insight into Le^b^ binding, we solved the crystal structure of BabA in complex with a hexasaccharide form of the Le^b^ antigen to 2.1-Å resolution (table S1). This oligosaccharide contains two fucose residues (Fuc1 and Fuc4), two galactose residues (Gal2 and Gal5), an *N*-acetylglucosamine residue (GlcNAc3), and a glucose residue (Glc6). Structure determination revealed a single, shallow binding site at the tip of the BabA crown (Fig. 2). All Le^b^ residues were visible in the electron density map except the terminal Glc6 residue, which is consequently not modeled (Fig. 2A). A comparison of the BabA crystal structure in the absence or presence of Le^b^ indicated that no conformational change occurred upon sugar binding [fig. S4; RMSD = 0.25Å for all Cα atoms (*22*)].

**Fig. 3:**
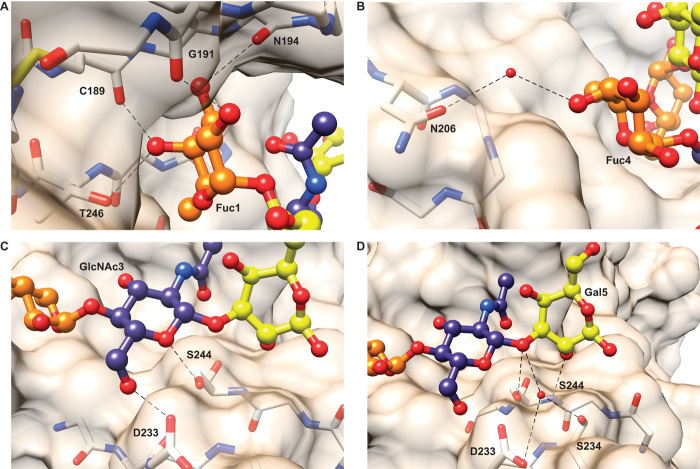
Interactions between BabA amino acids and Le^b^ residues. (**A**) Fuc1 forms hydrogen bonds with the carbonyl backbone of C189, G191, and N194 and the side chain of T246. (**B**) Fuc4 forms a water-mediated hydrogen bond with the N206 side chain. (**C**) GlcNAc3 forms hydrogen bonds with the D233 and S244 side chains. (**D**) Gal5 forms hydrogen bonds with the carbonyl backbone and side chain of S244. Gal5 also forms hydrogen bonds with a water molecule structured by the side chains of D233 and S234. Fucose, galactose, and *N*-acetylglucosamine residues are colored orange, yellow, and blue, respectively. Hydrogen bonds are represented by dotted black lines.

The crystallographic model indicated that binding is mediated by a network of hydrogen bonds between Le^b^ Fuc1, GlcNAc3, Fuc4, and Gal5 residues and a total of eight BabA amino acids. Fuc1 forms hydrogen bonds with the carbonyl backbone groups of C189, G191, and N194, and the hydroxyl group of the T246 side chain (Fig. 3A). Fuc4 interacts with the hydroxyl group of the N206 side chain through a water-mediated hydrogen bond (Fig. 3B). The GlcNAc3 residue forms two hydrogen bonds with the carboxyl and hydroxyl side-chain groups of D233 and S244, respectively (Fig. 3C). Last, Gal5 forms hydrogen bonds with both the S244 carbonyl backbone and the hydroxyl side-chain group. It also interacts with both the carboxyl group of the D233 side chain and the hydroxyl group of the S234 side chain through water-mediated hydrogen bonds (Fig. 3D). No interactions were observed between Gal2 and BabA.

**Fig. 4:**
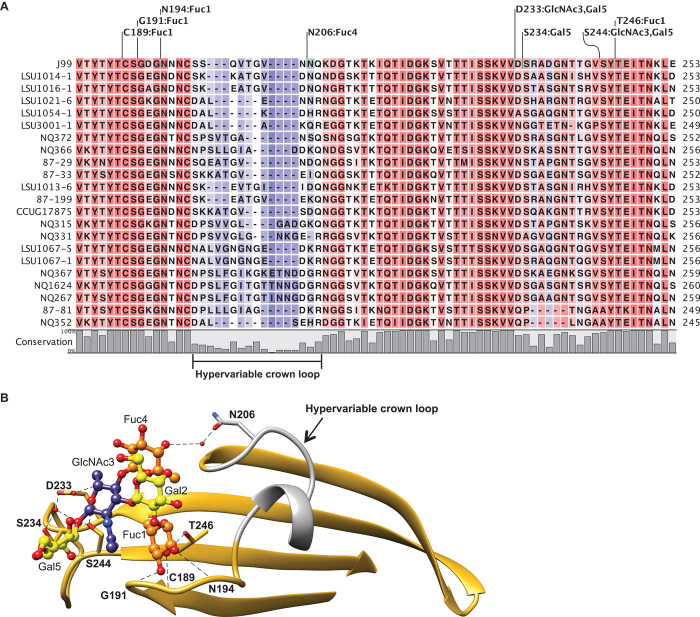
Conservation of the Le^b^ binding site of BabA. (**A**) Sequence alignment of the crown (residues 183 to 253) from BabA J99 with 21 *H. pylori* strains shown to bind Le^b^ glycoconjugates. Amino acids involved in hydrogen bond formation to each Le^b^ residue are indicated. (**B**) The crown β-strand unit is well conserved (gold) across the analyzed strains with the exception of the hypervariable crown loop (residues 198 to 207; gray). Fucose, galactose, and *N*-acetylglucosamine residues are colored orange, yellow, and blue, respectively. Hydrogen bonds are represented by dotted black lines.

Alignment of BabA J99, used in this study, with BabA from 21 *H. pylori* strains reported to bind Le^b^ glycoconjugates (*23*–*25*) revealed that the amino acids identified in mediating Le^b^ binding in our structural model are highly conserved with the exception of N206 (Fig. 4A). N206 is found within a region (residues 198 to 207 in BabA J99) with low amino acid conservation across the Le^b^ binding strains. We refer to this segment, which connects two antiparallel β-strands in the BabA J99 crown, as the hypervariable crown loop (Fig. 4B).

### Analysis of BabA:Le^b^ binding

**Fig. 5:**
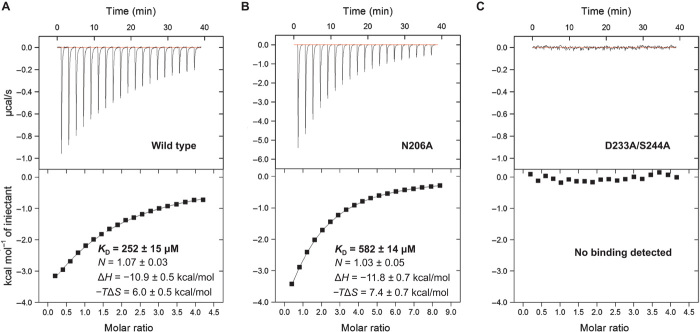
Binding affinity of wild-type and variant BabA proteins to Le^b^. (**A** and **B**) Calorimetric response (top) and binding isotherm (bottom) of (A) wild-type BabA titrated with Le^b^ and (B) the BabA N206A variant titrated with Le^b^. The continuous line in both lower panels represents the least-squares fit of the data to a single-site binding model. The reported thermodynamic parameters are the average (±SEM) of three independent experiments. (**C**) No calorimetric response (top) or binding isotherm (bottom) was obtained by titrating BabA containing D233A/S244A substitutions with Le^b^. All calorimetric titrations were performed at pH 7.4.

To further characterize the molecular interactions between BabA and Le^b^, we used isothermal titration calorimetry (ITC) to study binding at acidic and neutral pH (Fig. 5A and table S2). In agreement with the proposed model, binding was found to be a single-site interaction (*N* of ~0.91 and ~1.07 at pH 4.5 and 7.4, respectively). The thermodynamic parameters of the interaction indicate that binding is driven by noncovalent, that is, enthalpic contributions (Δ*H* of ~−12.2 and ~−10.9 kcal/mol at pH 4.5 and 7.4, respectively) rather than hydrophobic, that is, entropic contributions (−*T*Δ*S* of ~7.2 and ~6.0 kcal/mol at pH 4.5 and 7.4, respectively). Binding between BabA and Le^b^ molecules was observed to be a low-affinity interaction [*K*_D_ (dissociation constant) of ~227 and ~252 μM at pH 4.5 and 7.4, respectively]. There are no significant differences (*P* > 0.05) between the thermodynamic parameters and dissociation constants of BabA:Le^b^ binding at acidic and neutral pH.

To assess the significance of the observed interaction between the hypervariable crown loop and the Le^b^ Fuc4 residue, we generated and purified BabA with an N206A amino acid substitution. N206 was observed to form a single water-mediated hydrogen bond with Fuc4 through its polar side chain. In support of the structural model, we found that the BabA N206A variant had a lower affinity for Le^b^ (*K*_D_ of ~582 μM) (Fig. 5B). To assess the significance of the observed interactions between highly conserved amino acids in the crown and the Le^b^ GlcNAc3 and Gal5 residues, we generated and purified BabA with combined D233A and S244A amino acid substitutions. D233 and S244 both formed direct and water-mediated hydrogen bonds with GlcNAc3 and Gal5 through their polar side chains. In further support of our model, this BabA variant showed no detectable affinity for Le^b^ (Fig. 5C). None of the aforementioned amino acid substitutions caused a change in the global structure of BabA as determined by circular dichroism spectroscopy and differential scanning fluorimetry (fig. S5).

### Binding of BabA to related histo-blood group antigens

To investigate the relative importance of the Le^b^ sugar residues observed to interact with the crown, we studied the binding of BabA to a number of structurally similar fucosylated histo-blood group antigens by ITC (Table 1 and table S3). BabA bound with lower affinity (*K*_D_ ~617 μM) to the H-1 antigen, which lacks only the Le^b^ Fuc4 residue that forms a single water-mediated hydrogen bond with N206. No binding was observed between BabA and the Lewis^a^ (Le^a^) antigen, which lacks the Le^b^ Fuc1 residue that forms direct hydrogen bonds with C189, G191, N194, and T246. Furthermore, no binding was observed between BabA and the more distantly related Lewis^y^ (Le^y^) antigen and H-2 antigen. Although Le^y^ and H-2 contain the same residues that were observed in our structural model to bind BabA as Le^b^ and H-1, respectively, they differ by having a Galβ1-4GlcNAc glycosidic linkage. Finally, no binding was observed between BabA and SLe^x^. This glycan lacks the Le^b^ Fuc1 residue and contains a terminal *N*-acetylneuraminic acid residue adjoined to a Galβ1-4GlcNAc core. Histo-blood group antigens with Galβ1-3GlcNAc and Galβ1-4GlcNAc cores markedly differ in their conformational orientation (fig. S6).

**Table 1:**
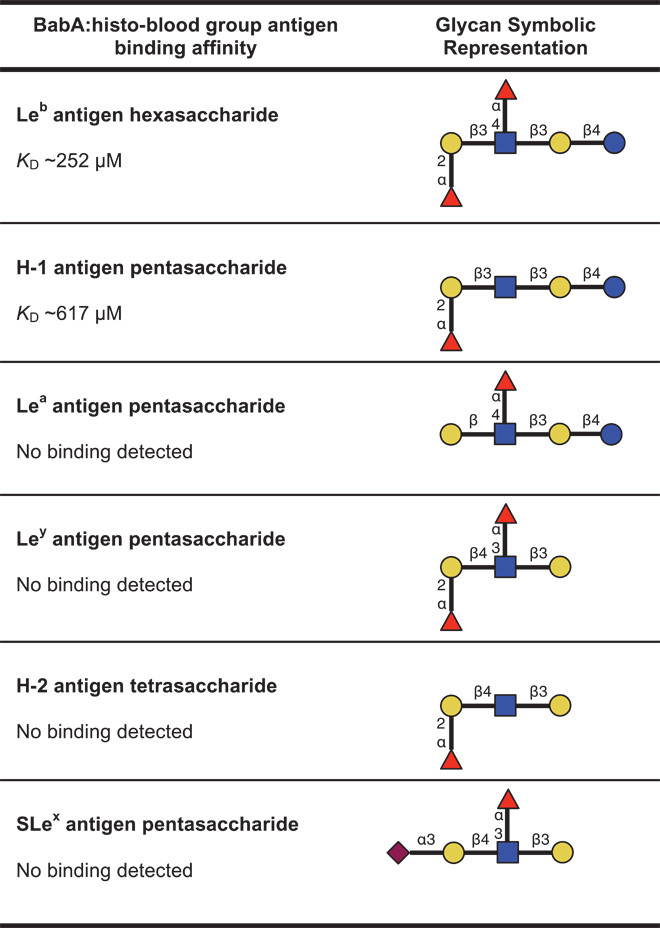
Binding affinity of BabA to various histo-blood group antigens. Glycan symbolic representations can be interpreted with the following key: fucose (Fuc, 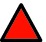), galactose (Gal, 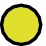), *N*-acetylglucosamine (GlcNAc, 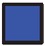), glucose (Glc, 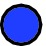), and *N*-acetylneuraminic acid (Neu5Ac, 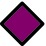). Binding affinity was determined through calorimetric titrations performed at pH 7.4.

## DISCUSSION

Here, we identified the molecular interactions involved in BabA-mediated *H. pylori* attachment to Le^b^ antigens. This was achieved by solving the crystal structure of the extracellular domain of BabA, from *H. pylori* strain J99, in the absence and presence of Le^b^. Upon comparison of the crystal structure of BabA to that of the functionally related SabA molecule, their striking similarity is immediately apparent. This can be considered to be an unexpected finding, given their low amino acid sequence identity, suggesting that their similar three-dimensional folds may be of functional significance. However, an in-depth analysis and understanding of the *H. pylori* outer leaflet is needed to aid the functional contextualization and rationalization of these adhesin structures. SabA does not contain the crown β-sheet motif of BabA, which we show has its Le^b^ binding site. This structural difference explains previous reports of the lack of affinity for SabA to Le^b^ (*20*). In line with previous studies, we also show that BabA has no affinity for SLe^x^ antigens, which are conversely recognized by SabA (*26*). Although a putative glycan binding pocket in SabA has been suggested, crystallographic insight into its structural basis for glycan recognition is needed to understand the differences that distinguish binding of SLe^x^ antigens between these two adhesins.

The Le^b^ binding interaction takes place in BabA at the edge of its crown region within a solvent-exposed binding site. The binding pocket is of a shallow topology, which is typically observed in carbohydrate binding proteins and is known to result in few ligand contacts (*27*). As a result, bacterial lectins, including SabA, typically display low glycan binding affinity (*20*, *28*). Low BabA:Le^b^ binding affinity was similarly found in our study and was additionally observed to be unaffected by acidic or neutral pH. This characteristic of BabA:Le^b^ binding may aid *H. pylori* colonization because these conditions are representative of the pH gradient found across the gastric mucus layer (*29*, *30*). Indeed, previous studies have demonstrated that *H. pylori* attachment to gastric mucin under neutral conditions (the pH of the deep gastric juxtamucosal layer) is mediated by the BabA:Le^b^ interaction (*31*–*33*). However, it must be noted that any potential effects of the membrane-spanning domain of BabA on Le^b^ binding affinity are not captured by this study. Furthermore, the multitude of BabA:Le^b^ interactions that occur between *H. pylori* and epithelium- or mucin-associated Le^b^ antigens would exponentially increase the binding affinity of a single bacterium during colonization. This effect of avidity on BabA:Le^b^ has been demonstrated in *H. pylori* J99, which binds Le^b^ glycoconjugates (which are multivalent complexes) with substantially higher affinity [*K*_A_ (association constant) of ~4 × 10^11^ M^−1^] (*24*).

Our structural model revealed that BabA J99 uses eight amino acids to bind to Le^b^. These amino acids are highly conserved with the exception of N206, which interacts with Le^b^ Fuc4 and is located within the hypervariable crown loop. The disparity in sequence identity and length observed in the hypervariable crown loop may result in modified secondary structure folds in other *H. pylori* strains and thereby affect the presentation of functional groups capable of interacting with Le^b^ Fuc4 or other residues. Thus, we speculate that the variability in this segment may be responsible, at least in part, for the differences seen in the affinity of *H. pylori* strains toward Le^b^ antigens (*24*). Indeed, the interaction between N206 and Fuc4 did have an effect on determining the binding affinity of BabA:Le^b^ in this study. We found that an alanine point substitution at position 206 resulted in a ~2.3-fold reduction in binding affinity. Furthermore, analysis of the binding interaction between BabA and the H-1 antigen, the other type 1 histo-blood group antigen known to act as a receptor for BabA in individuals with the blood group O phenotype (*24*), supported this finding. H-1 lacks only the Fuc4 residue of Le^b^ and binds to BabA with ~2.4-fold lower affinity than Le^b^. Thus, our data suggest that the interaction between the BabA crown and Le^b^ Fuc4 can play a substantive role in determining binding affinity.

Intriguingly, no DNA or protein sequences with similarity to the BabA crown were identified in *H. pylori* or any other organism. In this light, the crown can be considered unique to BabA to enable attachment of *H. pylori* to specific histo-blood group antigens in the human gastric mucosa. This is achieved through the network of hydrogen bonds presented because our data show that partial modifications of these BabA:Le^b^ interactions result in a loss of recognition. As observed through the analysis of the BabA:Le^a^ interaction, the absence of only the Fuc1 residue from Le^b^ results in a complete loss of binding. This occurs despite possible interactions between the crown and the other sugar residues found in Le^b^. However, BabA contacts with Fuc1, though necessary, are not sufficient to confer glycan recognition. Combined alanine point substitutions to BabA at D233 and S244, which form direct and water-mediated hydrogen bonds through their side chains to GlcNAc3 and Gal5, also result in a complete loss of binding to Le^b^. This occurs despite possible interactions between the crown and Fuc1, and also Fuc4. Thus, we conclude that no single sugar residue is responsible for Le^b^ recognition by BabA. Rather, it is the network of hydrogen bonds to multiple residues that forms the basis of molecular recognition. Consequently, it is no surprise that BabA does not bind to the distantly related Le^y^ and H-2 antigens because they have completely different three-dimensional conformations. In most of the Western population, Le^a^, Le^y^, and H-2 antigens are not found in the gastric mucus layer, and it is known that they do not act as receptors for *H. pylori* (*6*, *8*, *34*).

The structural model presented in this study explains the basis of BabA-mediated *H. pylori* attachment to Le^b^ antigens. The recognition of H-1 antigens, which act as host receptors for BabA in blood group O individuals, is similarly rationalized through this work. However, further investigation is needed to understand the molecular basis underlying the contrasting differences exhibited by various *H. pylori* strains in the recognition of the different histo-blood group antigens that act as host receptors for BabA in blood group A, B, and O individuals (*24*). A full comprehension of the molecular interactions required for adhesion is a promising lead for the development of new strategies for the treatment of *H. pylori* infections.

## METHODS

### Study design

The objective of this study was to identify the molecular interactions that mediate the binding of BabA to Le^b^ antigens. To achieve this, we used x-ray crystallography to study the interactions between BabA and Le^b^. The BabA molecule used in this study was the extracellular domain of BabA from *H. pylori* J99 [a strain that has been shown to bind Le^b^ antigens (*24*)] recombinantly expressed in the periplasmic space of *E. coli*. A commercially available hexasaccharide form of Le^b^ was used in this study. A variety of established biophysical, biochemical, and spectroscopic techniques were used to probe and validate the structural model.

### Cloning, expression, and purification of BabA proteins

A gene fragment encoding the predicted N-terminal extracellular domain (amino acids 1 to 527) of mature BabA was amplified from *H. pylori* J99 genomic DNA (donated by J. Atherton, University of Nottingham) with KOD DNA polymerase (Novagen), using the primers specified in table S4, and cloned into the pOPE101 expression vector (Progen Biotechnik). The *E. coli* XL10 Gold strain (Agilent Technologies) was used for both vector construction and protein expression. Transformed cells were grown in lysogeny broth [supplemented with ampicillin (100 μg/ml), tetracycline (12.5 μg/ml), and 0.1 M glucose] at 37°C until the OD_600_ (optical density at 600 nm) reached ~0.6. Protein expression was induced through the addition of isopropyl β-d-1-thiogalactopyranoside (IPTG) to a final concentration of 0.1 mM for 16 hours at 24°C. BabA was harvested from the periplasmic space using an osmotic shock procedure as described in (*7*). Recombinant BabA, containing amino acids 10 to 527 of mature BabA in addition to three C-terminal polypeptide tags (6xLys-c-Myc-6xHis), was purified through immobilized metal ion (nickel) affinity chromatography and size exclusion chromatography (yield: 1 to 2 mg/liter of bacterial culture) (*7*).

Selenomethionine-substituted BabA (SeMet BabA) was generated using the same expression construct and *E. coli* strain as above. Cells were grown in M9 minimal medium supplemented with thiamine (2 mg/liter), glucose (4 g/liter), 2 mM MgSO_4_, 0.1 mM CaCl_2_, ampicillin (100 μg/ml), and tetracycline (12.5 μg/ml) at 37°C until the OD_600_ reached ~0.6. The following amino acids were added for 15 min before IPTG induction at 24°C for 16 hours: lysine, phenylalanine, and threonine (100 mg/liter each) and isoleucine, leucine, valine, and dl-selenomethionine (50 mg/liter each). Purification was performed under the same conditions as the unlabeled protein.

Expression constructs encoding BabA variants containing either N206A or D233A/S244A substitutions were generated with a Phusion site-directed mutagenesis kit (Thermo Scientific), using the primers shown in table S4. Expression and purification was performed under the same conditions as the wild-type unlabeled protein.

### Crystallization

Apo crystals of BabA were obtained by sitting drop vapor diffusion by mixing equal volumes of protein solution (20 mg/ml) and well solution [22% polyethylene glycol (PEG) 3350, 0.2 M ammonium acetate, and 0.1 M sodium citrate (pH 5.6)]. Cocrystals of BabA were similarly obtained using a well solution of 22% PEG 3350 and 0.1 M sodium propionate, sodium cacodylate trihydrate, bis-tris propane (PCTP; pH 6.0). The complex was formed by adding a fourfold molar excess of Le^b^ antigen hexasaccharide (IsoSep AB) and preincubating at 4°C for 30 min before dispensing trials. Both crystals appeared after 3 days and continued to grow for a further 10 days at 20°C.

SeMet crystals were obtained by sitting drop vapor diffusion by mixing 250 nl of protein (20 mg/ml), 250 nl of well solution [34% PEG 3350, 0.2 M magnesium acetate, 0.1 M PCTP broad range buffer (pH 6.0)], and 50 nl of seed stock. The seed stock was prepared by placing three large apo crystals into a microcentrifuge tube with 50 μl of reservoir solution. A seed bead was then added and vortexed for 2 min immediately before dispensing plates. Crystals appeared overnight and continued to grow for a further 5 days at 20°C. Crystals were cryoprotected by transfer to a well solution including 20% glycerol for 30 s, plunged into liquid nitrogen, and kept at 100 K during data collection.

### Structure solution

Highly redundant Se SAD data were remotely collected at the European Synchrotron Radiation Facility (ESRF), Grenoble, France (Beamline ID23-1, DECTRIS Pilatus detector, 100 K) and processed using XDS, Truncate, and Aimless (*35*). The anomalous completeness of the data was 98.3% (87.2% in outer shell), with an anomalous multiplicity of 12.0 overall (7.0 in outer shell). Anomalous correlation between half-sets was 0.760 in the inner shell and 0.280 overall.

Crank2 (CCP4i) (*36*–*42*) was used to solve the structure. The programs used in pipeline were ShexlC (*37*), ShelxD (*37*), Refmac5 (*38*), Solomon (*39*), Multicomb (*40*), Parrot (*41*), and Buccaneer (*42*). ShelxC found four selenium atoms. Eighty-nine percent of the residues were built with four gaps in the chain. Coot (*43*) was used for model building; rebuilding of chains across gaps was necessary where they had crossed to symmetry-related molecules in the initial model. The model was further refined as a 1.9-Å data set (Refmac5), although the data were weak in the outer shell. The residues built in the structure run from Q27 to L527 (fig. S7) with two loops missing from the sequence (between A282–P291 and S402–Q410). The Ramachandran plot showed that 100% of residues were allowed with 96.04% in the preferred region.

This apo model was used in molecular replacement [Phaser (*44*)] to solve the structure of the protein complexed with the sugar. X-ray data for the complex were collected at Diamond Light Source (DLS), Didcot, UK (Beamline i04, DECTRIS Pilatus detector, 100 K). The sugar was fitted using Coot (0.8) (*43*), and refinement was again carried out using Refmac5. Protein residues Q27 to K528 were modeled in the density, again with two gaps in chain (between A282–F293 and S402–Q410). Five of the six sugar units were modeled; the terminal glucose (Glc6) was not seen and density for the adjoining galactose (Gal5) was partial. X-ray data and refinement statistics are shown in table S1.

### Isothermal titration calorimetry

Calorimetric measurements were performed at 25°C on a MicroCal iTC200 System (GE Healthcare) in a buffer containing 20 mM tris-Cl (pH 7.4) and 300 mM NaCl or 50 mM KH_2_PO_4_ (pH 4.5). The sample cell was filled with protein at a concentration of 0.1 mM and stirred at 1000 rpm until the system reference power was equilibrated to 6 μcal/s. The injection syringe contained histo-blood group antigen at a concentration of 2 mM, and 19 repeated 2-μl injections were made, allowing 120 s between each titration. These conditions were modified when studying the BabA:H-1 and BabA N206A variant:Le^b^ interactions, where the protein and oligosaccharide concentrations were increased to 0.25 and 10 mM, respectively, to attain a larger calorimetric response. NITPIC (*45*) was used for baseline autodetermination, and calorimetric data were analyzed by peak integration using ORIGIN 7.0 software. All histo-blood group antigens (purity >90%) were obtained from Elicityl SA, except Le^b^, which was obtained from IsoSep AB.

### Sequence analysis

For identification of proteins with structural similarity to BabA, the apo-BabA PDB file (accession code: 4ZH0) was uploaded onto the Dali server (*21*) for an atomic coordinates similarity search within the PDB. For analysis of strain conservation of the Le^b^ binding site, available *babA* gene sequences corresponding to the crown (amino acids 183 to 253 in mature BabA J99) from Le^b^ binding strains identified in (*23*–*25*) were obtained from the European Nucleotide Archive and GenBank. After removal of duplicate sequences, CLC Main WorkBench 7.6 (CLC bio) was used for the multiple sequence alignment (protein) of BabA J99 with 21 *H. pylori* strains.

To search for DNA sequences with similarity to the BabA crown, the BabA gene fragment corresponding to the crown [that is, complement(914203–914421) in GenBank accession no. AE001439.1] was submitted to Nucleotide-BLAST. The nucleotide collection (nr/nt) database was searched using megablast, discontiguous, and blastn algorithms. To search for protein sequences with similarity to the BabA crown, the BabA protein fragment corresponding to the crown [that is, residues 203 to 273 in GenBank accession no. AAD06409.1] was submitted to Protein-BLAST. The nonredundant protein sequences (nr) database was searched using BLAST algorithms optimized for protein-protein, position-specific iterated, pattern hit initiated, and domain enhanced lookup time accelerated BLAST.

### Statistical analysis

For crystallographic experiments, apo-BabA and BabA:Le^b^ structural models were built from diffraction data sets collected from single crystals after multiple rounds of iterative refinement. For biophysical analysis, binding isotherms obtained from ITC were fitted to a nonlinear least squares curve with Origin 7.0 after multiple iterations until a fixed minimal χ^2^ value was achieved. An unpaired two-tailed Welch’s *t* test was used to determine if the thermodynamic parameters associated with BabA:Le^b^ binding from three independent experiments at pH 4.5 and 7.4 were significantly different from each other.

## Supplementary Material

http://advances.sciencemag.org/cgi/content/full/1/7/e1500315/DC1

## SUPPLEMENTARY MATERIALS

Supplementary material for this article is available at http://advances.sciencemag.org/cgi/content/full/1/7/e1500315/DC1 Materials and Methods Fig. S1. Glycan symbolic representations of the fucosylated histo-blood group antigens that act as BabA receptors. Fig. S2. Schematic illustration of the predicted domain structure of BabA. Fig. S3. Alignment of BabA J99 and SabA 26695 protein sequences annotated with secondary structure elements. Fig. S4. Superimposition of BabA from apo and cocrystal structures. Fig. S5. Secondary structure and thermal stability of BabA and BabA variants. Fig. S6. Type 1 and type 2 fucosylated histo-blood group antigen molecular models. Fig. S7. Rainbow representation of apo-BabA. Table S1. X-ray diffraction data collection and refinement statistics. Table S2. Thermodynamic parameters of BabA:Le^b^ interaction at pH 4.5 and 7.4. Table S3. Binding affinity of BabA to various histo-blood group antigens. Table S4. Oligonucleotides used in BabA cloning and site-directed mutagenesis. References (*6*, *22*, *46*, *47*)
