# Supplementary material for: Structural basis of Lewisb antigen binding by the Helicobacter pylori adhesin BabA
Source: Sci Adv. 2015 Aug 14;1(7):e1500315. doi: 10.1126/sciadv.1500315 (PMC4643811; doi:10.1126/sciadv.1500315)
Supplement: http://advances.sciencemag.org/cgi/content/full/1/7/e1500315/DC1 [file supp_1_7_e1500315__index.html]

Science Advances | Science Advances

## Supplementary Materials

**This PDF file includes:**

- Materials and Methods
- Fig. S1. Glycan symbolic representations of the fucosylated histo-blood group antigens that act as BabA receptors.
- Fig. S2. Schematic illustration of the predicted domain structure of BabA.
- Fig. S3. Alignment of BabA J99 and SabA 26695 protein sequences annotated with secondary structure elements.
- Fig. S4. Superimposition of BabA from apo and cocrystal structures.
- Fig. S5. Secondary structure and thermal stability of BabA and BabA variants.
- Fig. S6. Type 1 and type 2 fucosylated histo-blood group antigen molecular models.
- Fig. S7. Rainbow representation of apo-BabA.
- Table S1. X-ray diffraction data collection and refinement statistics.
- Table S2. Thermodynamic parameters of BabA:Leb interaction at pH 4.5 and 7.4.
- Table S3. Binding affinity of BabA to various histo-blood group antigens.
- Table S4. Oligonucleotides used in BabA cloning and site-directed mutagenesis.
- References (*6, 22, 46, 47*)

Download PDF

**Files in this Data Supplement:**

- Adobe PDF - 1500315\_SM.pdf
