# Supplementary material for: Structural basis of Lewisb antigen binding by the Helicobacter pylori adhesin BabA
Source: Sci Adv. 2015 Aug 14;1(7):e1500315. doi: 10.1126/sciadv.1500315 (PMC4643811; doi:10.1126/sciadv.1500315)
Supplement: http://advances.sciencemag.org/cgi/content/full/1/7/e1500315/DC1 [file 1500315_SM.pdf]

Science Advances

AAAS

www.advances.sciencemag.org/cgi/content/full/1/7/e1500315/DC1

Supplementary Materials for

Structural basis of Lewis^{b} antigen binding by the *Helicobacter pylori* adhesin BabA

Naim Hage, Tina Howard, Chris Phillips, Claire Brassington, Ross Overman, Judit Debreczeni, Paul Gellert, Snow Stolnik, G. Sebastiaan Winkler, Franco H. Falcone

Published 14 August 2015, *Sci. Adv.* **1**, e1500315 (2015)
DOI: 10.1126/sciadv.1500315

This PDF file includes:

Materials and Methods
Fig. S1. Glycan symbolic representations of the fucosylated histo-blood group antigens that act as BabA receptors.
Fig. S2. Schematic illustration of the predicted domain structure of BabA.
Fig. S3. Alignment of BabA J99 and SabA 26695 protein sequences annotated with secondary structure elements.
Fig. S4. Superimposition of BabA from apo and cocrystal structures.
Fig. S5. Secondary structure and thermal stability of BabA and BabA variants.
Fig. S6. Type 1 and type 2 fucosylated histo-blood group antigen molecular models.
Fig. S7. Rainbow representation of apo-BabA.
Table S1. X-ray diffraction data collection and refinement statistics.
Table S2. Thermodynamic parameters of BabA:Le^{b} interaction at pH 4.5 and 7.4.
Table S3. Binding affinity of BabA to various histo-blood group antigens.
Table S4. Oligonucleotides used in BabA cloning and site-directed mutagenesis.
References (*6*, *22*, *46*, *47*)

Supplementary Materials and Methods

Secondary structure analysis

Circular dichroism spectra of BabA and BabA variants (containing N206A and D233A/S244A substitutions) was measured using a J-810 Spectropolarimeter (Jasco). Protein concentration was 1 μM in a buffer containing 20 mM tris-Cl (pH 7.4) and 3 mM NaCl. Measurements were made at 25°C in a quartz cell with a 0.1 cm pathlength at a data pitch of 0.5 nm and scanning speed of 100 nm/min. Reported spectra are baseline-corrected for buffer alone and averaged from three independent scans.

Thermal stability analysis

For differential scanning fluorimetry experiments, SYPRO Orange dye (20× final concentration) was added to 10 μM BabA and BabA variants (containing N206A and D233A/S244A substitutions) in a buffer containing 20 mM Tris-Cl (pH 7.4) and 300 mM NaCl. Changes in fluorescence were measured across an increasing temperature gradient from 25°C to 60°C using a LightCycler 480 II (Roche) at a ramp rate of 0.01^{o}C/s. Primary data points from three independent experiments were fitted to a 6-parameter unfolding equation *(46)* using the Prism analysis package (GraphPad Software).

Supplementary Figures and Tables

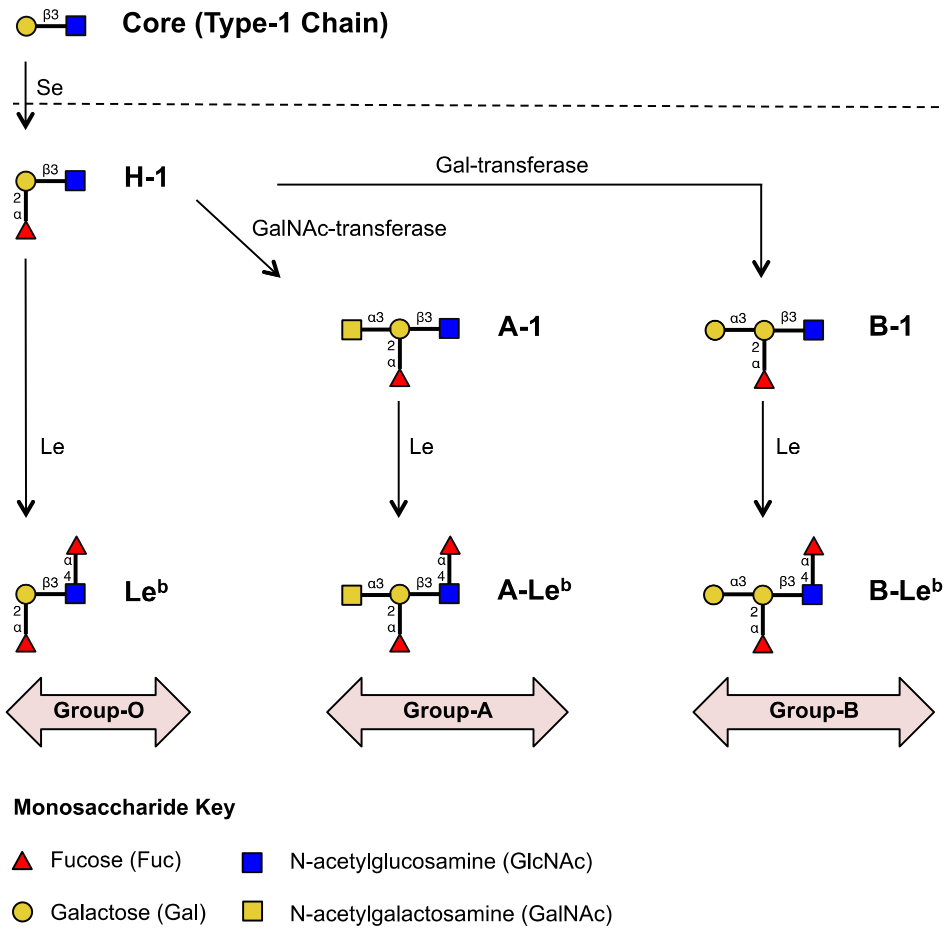

Fig. S1. Glycan symbolic representations of the fucosylated histo-blood group antigens that act as BabA receptors. The terminal glycan epitopes of the secreted ABH/Lewis antigens that act as receptors for BabA in the gastric mucosa are shown. Histo-blood group antigens are produced after the fucosylation of a type 1 lacto series core chain by an α1,2-fucosyltransferase (Se), which generates the H-1 antigen. This oligosaccharide is modified by a GalNAc-transferase in blood group A individuals and Gal-transferase in blood group B individuals, thereby producing the A-1 and B-1 antigens, respectively. All ABH antigens are subjected to further fucosylation by an α1,3/4-fucosyltransferase (Le), which produces Le^b, A-Le^b and B-Le^b in blood group O, A and B individuals, respectively *(6)*.

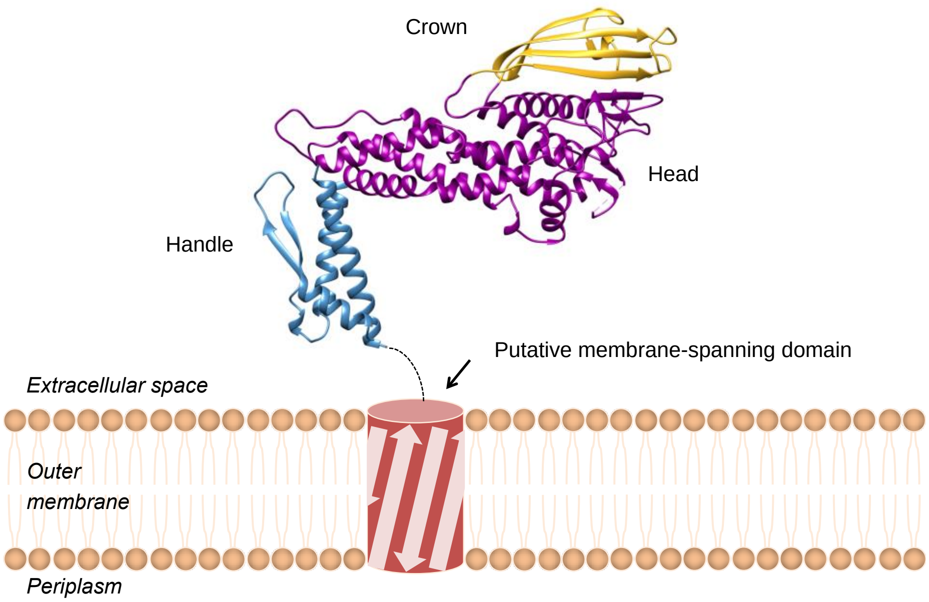

Fig. S2. **Schematic illustration of the predicted domain structure of BabA.** Indicated are the handle (blue) and head regions (dark magenta), and the crown β-strand unit (gold) of the extracellular domain. BabA is anchored to the *H. pylori* outer membrane through a putative C-terminal transmembrane β-barrel domain.

```
                                         α-N
BabA
SabA
             1                                                          60
BabA J99     EDDGFYTSVGYQIGEAAQMVTNTKGIQDLSDRYESLNNLLNRYSTLNTLIKLSADPSAIN
SabA 26695   EDNGFFVSAGYQIGEAVQMVKNTGELKNLNEKYEQLSQYLNQVASLKQSIQNANNIELVN
             1                                                          60
             **:**:.*.*******.***.**  :::*.::**.*.: **: ::*:  *: : : . :*

                     α-1
BabA
SabA
             61                                                        118
BabA J99     AVRENLGASAKNLIGDKANSPAYQAVLLAINAAVGFWNVVG--YVTQCGGNANGQKSISS
SabA 26695   SSLNYLKSFTNNNYNSTTQSPIFNAVQAVITSVLGFWSLYAGNYFTFFVGKKVGDSGQ--
             61                                                        118
             :  : * : ::*   ..::** ::**  .*.:.:***.: .   *.*   *:  *:..

                   β-1                               α-1a
BabA
SabA
             119                                                       178
BabA J99     KTIFNNEPGYRSTSITCSLNGHSPGYYGPMSIENFKKLNEAYQILQTALKRGLPALKENN
SabA 26695   PASVQGNPPFKTIIENCSGIE--NCAMDQTTYDKMKKLAEDLQAAQTN------------
             119                                                       164
              : .: :* :::   .**             : :::*** *  *   **

                                              β-2
BabA
SabA
             179                                                       238
BabA J99     GKVNVTYTYTCSGDGNNNCSSQVTGVNNQKDGTKTKIQTIDGKSVTTTISSKVVDSRADG
SabA 26695   ----------SATKGNNLCALSGCAATDSTSN--PPNSTVS---NALNLAQQLMDLIANT
             165                                                       209
                       .: .*** *: .  ...:...      .*:.    : .::.:::*  *:

                                    α-1b
BabA
SabA
             239                                                       298
BabA J99     NTTGVSYTEITNKLEGVPDSAQALLAQASTLINTINNACPYFHASNSSEANAPKFSTTTG
SabA 26695   -KTAMMWKNI--VISGVSNTSGAIT--------S------------------TNYPT---
             210                                                       237
              .*.: :.:*   :.** ::: *:           :                   :: *
```

```
                           α-2
BabA
SabA
                299                                                       358
BabA J99        KICGAFSEEISAIQKMITDAQELVNQTSVINEHEQTTPVGNNNGKPFNPFTDASFAQGML
SabA 26695      -QYAVF-NNIKAMIPILQQAVTLSQSNHTLSASLQAQATGSQ--------TNPKFAKDIY
                238                                                       287
                   ..* ::*.*:  :: :*  * :.. .:.   *:  .*.:        *: .**: :

                   α-3
BabA
SabA
                359                                                       418
BabA J99        ANASAQAKMLNLAEQVGQAINPERLSGTFQNFVKGFLATCNNPSTAGTGGTQGSAPGTVT
SabA 26695      TFAQNQKQVISYAQDIFNLFNS-IPAEQYKYLEKAYLKIPNA----------GSTPTNP-
                288                                                       335
                : *. * :::. *::: : :*    :  :: : *.:*   *           **:* .

                                         α-4                         α-C1
BabA
SabA
                419                                                       478
BabA J99        TQTFASGCAYVGQTITNLKNSIAHFGTQEQQIQQAENIADTLVNFKSRYSELGNTYNSIT
SabA 26695      ---YR-QVVNLNQEVQTIKNNVSYYGNRV---DAALSVARDVYNLKSNQAEIVTAYNDAK
                336                                                       388
                   :    . : * : .:**.::::*.:    : * .:*  : *:**. :*: .:**. .

                                           β-C              α-C2
BabA
SabA
                479                                                       534
BabA J99        ---TALSNIPNAQSLQN-AVSKKNNPYSPQGIDTNYYLNQNSYNQIQTINQELGRNPFRK
SabA 26695      TLSEEISKLPHNQVNTKDIVTLPYDKNAPAAGQSNYQINPEQQSNLNQALAAMSNNPFKK
                389                                                       448
                     :*::*. *   :  *:   :  :* . ::** :* :. .:::     :..***:*

                                           Conserved domain within
BabA
SabA
                535                                                       594
BabA J99        VGIVSSQTNNGAMNGIGIQVGYKQFFGQKRKWGARYYGFFDYNHAFIKSSFFNSASDVWT
SabA 26695      VGMISSQNNNGALNGLGVQVGYKQFFGESKRWGLRYYGFFDYNHGYIKSSFFNSSSDIWT
                449                                                       508
                **::***.****:**:*:*********:.::** **********.:********:**:**
```

```
                   Helicobacter outer membrane protein superfamily
BabA
SabA
           595                                                        654
BabA J99   YGFGADALYNFINDKATNFLGKNNKLSVGLFGGIALAGTSWLNSEYVNLATMNNVYNAKM
SabA 26695 YGGGSDLLVNIINDSI---TRKNNKLSVGLFGGIQLAGTTWLNSQYVNLTAFNNPYSAKV
           509                                                        565
           ** *:* * *:***.      ************* ****:****:****:::** *.**:

                  corresponding to putative transmembrane domain
BabA
SabA
           655                                                        714
BabA J99   NVANFQFLFNMGVRMNLARPKKKDSDHAAQHGIELGLKIPTINTNYYSFMGAELKYRRLY
SabA 26695 NATNFQFLFNLGLRTNLATARKKDSEHSAQHGIELGIKIPTITTNYYSFLGTQLQYRRLY
           566                                                        625
           *.:*******:*:* ***  :****:*:********:*****.******:*::*:*****


BabA
SabA
           715     724
BabA J99   SVYLNYVFAY
SabA 26695 SVYLNYVFAY
           626     635
           **********
```

Fig. S3. Alignment of BabA J99 and SabA 26695 protein sequences annotated with secondary structure elements. Full-length BabA and SabA share 40% sequence identity and 24% sequence similarity. The extracellular domains of BabA and SabA share 26% sequence identity and 28% sequence similarity. The transmembrane domains of BabA and SabA share 73% sequence identity and 15% sequence similarity. Indicated are positions that have a fully conserved residue (*****); conservation between groups with strongly similar properties (> 0.5 in the Gonnet PAM 250 matrix) (**:**); conservation between groups with weakly similar properties (< 0.5 in the Gonnet PAM 250 matrix) (**.**). Alignment was performed with Clustal Omega.

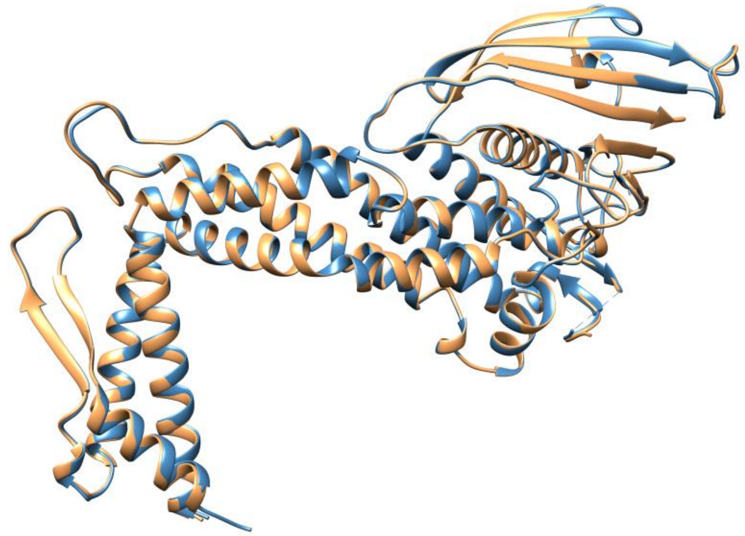

Fig. S4. Superimposition of BabA from apo and cocrystal structures. No global conformational change occurs in BabA (sandy brown) after Le^{b} complex formation (steel blue) - RMSD = 0.25Å for all Cα atoms *(22)*.

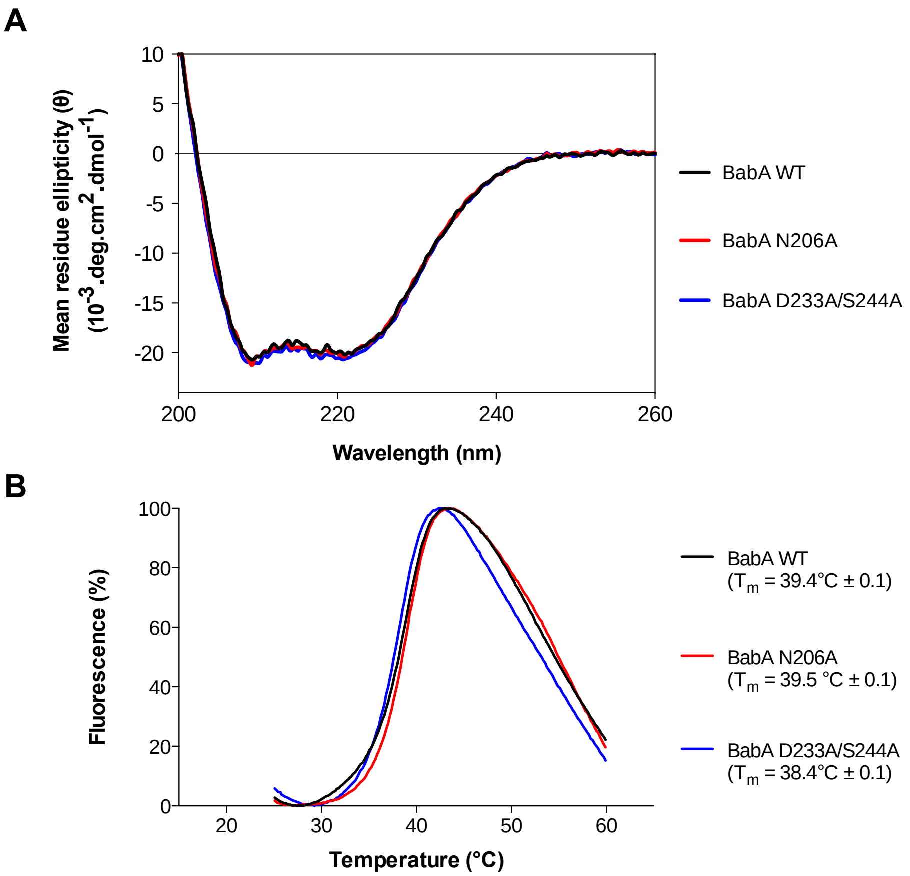

Fig. S5. Secondary structure and thermal stability of BabA and BabA variants. (**A**) Overlay of far-UV circular dichroism spectra averaged from three independent experiments. (**B**) Temperature-induced unfolding transition determined using differential scanning fluorimetry. The reported midpoint temperature of each protein unfolding transition (T_m) is the average (± SEM) from three independent experiments.

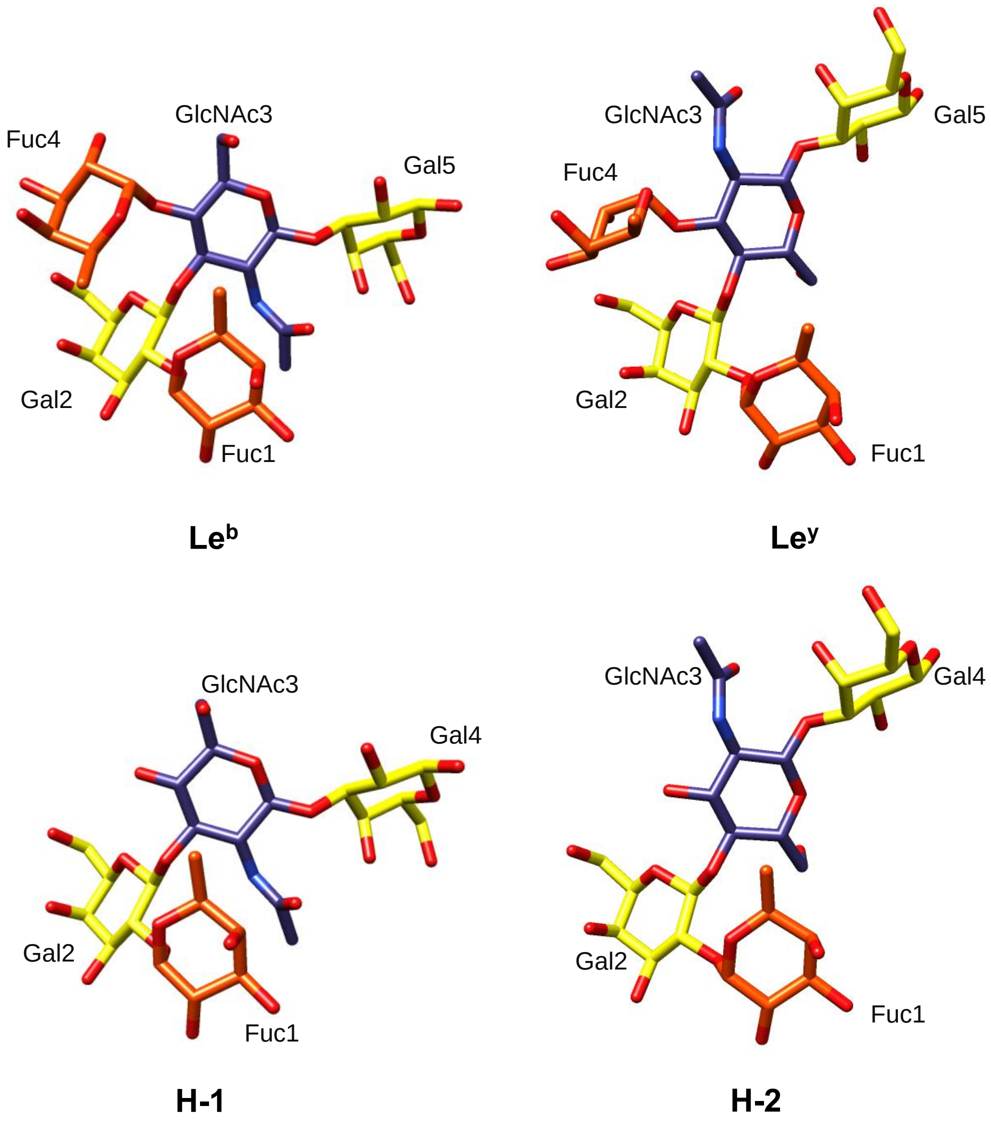

Fig. S6. Type 1 and type 2 fucosylated histo-blood group antigen molecular models. Stick models of Le^b and H-1 (type 1) antigens show a distinctly different three-dimensional orientation to Le^y and H-2 (type 2) antigens due to Galβ1-3GlcNAc and Galβ1-4GlcNAc linkages, respectively. Models were calculated in minimum energy conformations with the SWEET-II system *(47)*. Fucose, galactose and *N*-acetylglucosamine residues are colored orange, yellow and blue, respectively.

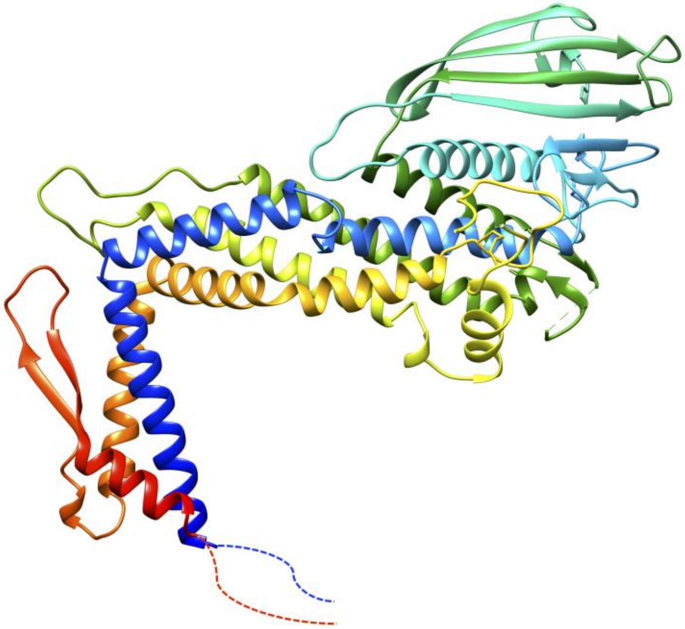

Fig. S7. Rainbow representation of apo-BabA. Recombinant BabA used in this study contains amino acids 10 to 527 of mature BabA followed by three C-terminal polypeptide tags (6xLys-c-Myc-6xHis). The residues visible in the apo-BabA electron density map run from 27-527. BabA amino acids 10-26 (blue dotted line) and the C-terminal polypeptide tags corresponding to amino acids 528-552 (red dotted line) were not modeled.

Table S1. X-ray diffraction data collection and refinement statistics.

**Components**	SeMet BabA (SAD data collection)	BabA:Le^b
**Data collection**		
Space group	P 21 21 21	P 21 21 21
Cell dimensions		
a, *b*, *c* (Å)	60.83 93.04 96.92	60.59 91.77 96.42
α, β, γ (°)	90 90 90	90 90 90
Wavelength (Å)	0.97925 (PEAK)	0.920
Resolution (Å)	48.46-1.91 (1.98-1.91)	44.78-2.12 (2.18-2.12)
$R_{\text{merge (all I+ and I-)}}$	0.236 (3.427)	0.141 (0.643)
$I/\sigma I$	13.2 (0.9)	11.8 (3.0)
Completeness (%)	98.7 (89.2)	99.1 (99.4)
Multiplicity[1]	23.1 (13.8)	6.6 (6.5)
**Refinement**		
Resolution (Å)	48.46-1.91 (1.98-1.91)	44.78-2.12 (2.18-2.12)
No. reflections	39877 (96.7%)	29319 (98.9%)
$R_{\text{work/}}$ R_{free}	0.189/0.231 (0.378/0.331)	0.171/0.223 (0.224/0.271)
No. atoms		
Protein	3662	3654
Ligand/ion	-	57
Water	132	156
B-factors		
Protein	25.327	20.97
Ligand/ion		35.413 (31.9)[§]
Water	34.124	30.034
R.m.s deviations		
Bond lengths (Å)	0.0184	0.0165
Bond angles (°)	1.7756	1.726

[§]Excluding the partially visible galactose (Gal5) moiety

Table S2. Thermodynamic parameters of BabA:Le^{b} interaction at pH 4.5 and 7.4. The upper panels in each ITC trace show a representative calorimetric response obtained by titrating BabA with Le^{b}. The lower panels depict the binding isotherm obtained where the continuous line represents the least-squares fit of the data to a single-site binding model. The reported thermodynamic parameters are the average (± SEM) of three independent experiments. There are no significant differences between the thermodynamic parameters and dissociation constants of BabA:Le^{b} binding at pH 4.5 and 7.4 (unpaired two-tailed Welch's t-test, $P > 0.05$).

Thermodynamic parameters	ITC trace
**pH = 4.5**<br>$K_D = 227 \pm 22$ μM<br>$N = 0.91 \pm 0.15$<br>$\Delta H = -12.2 \pm 1.8$ kcal/mol<br>$-T\Delta S = 7.2 \pm 1.8$ kcal/mol	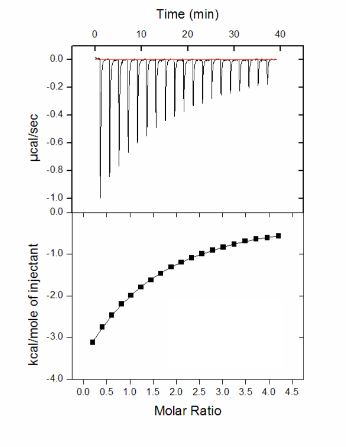
**pH = 7.4**<br>$K_D = 252 \pm 15$ μM<br>$N = 1.07 \pm 0.03$<br>$\Delta H = -10.9 \pm 0.5$ kcal/mol<br>$-T\Delta S = 6.0 \pm 0.5$ kcal/mol	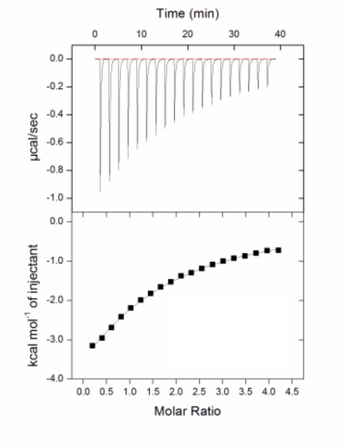

Table S3. Binding affinity of BabA to various histo-blood group antigens. Glycan structural representations can be interpreted with the following key: fucose (Fuc,), galactose (Gal,), *N*-acetylglucosamine (GlcNAc,), glucose (Glc,), *N*-acetylneuraminic acid (Neu5Ac,). The upper panels in each ITC trace show a representative calorimetric response obtained by titrating BabA with the respective histo-blood group antigens. The lower panels depict the binding isotherm obtained where the continuous line represents the least-squares fit of the data to a single-site binding model (where applicable). Calorimetric titrations were performed at pH 7.4.

Histo-blood group antigen and binding affinity	ITC trace
**Le^{b} antigen (hexasaccharide form)**<br>Fucα1-2Galβ1-3(Fucα1-4)GlcNAcβ1-3Galβ1-4Glc<br>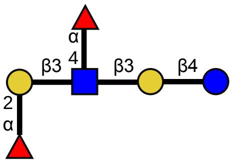<br><br>K_D = 252 µM[a]	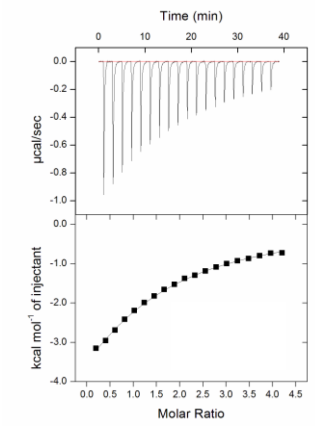
**H-1 antigen (pentasaccharide form)**<br>Fucα1-2Galβ1-3GlcNAcβ1-3Galβ1-4Glc<br>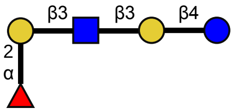<br><br>K_D = 617 µM[b]	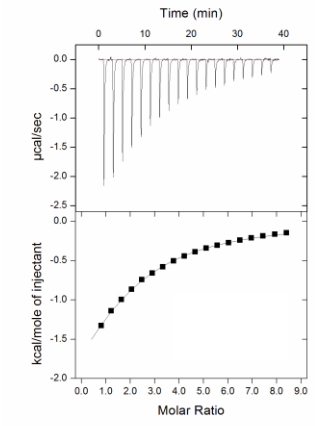

Le^{a} antigen (pentasaccharide form)
Galβ1-3(Fucα1-4)GlcNAcβ1-3Galβ1-4Glc

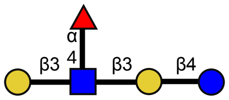

No binding detected

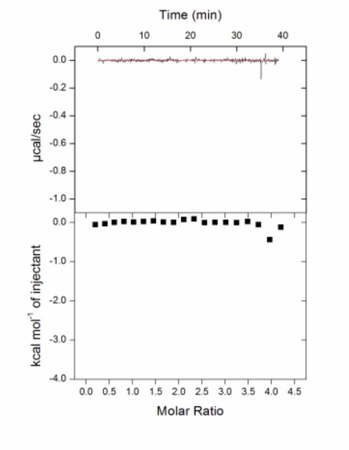

Le^{y} antigen (pentasaccharide form)
Fucα1-2Galβ1-4(Fucα1-3)GlcNAcβ1-3Gal

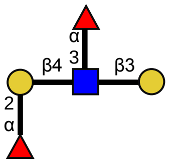

No binding detected

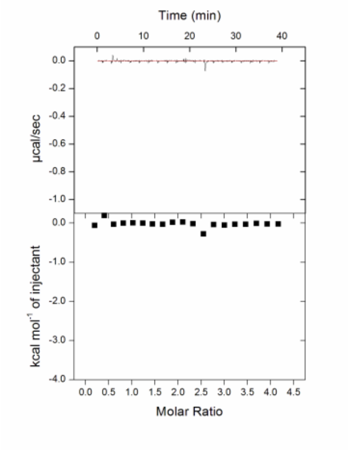

H-2 antigen (tetrasaccharide form)
Fucα1-2Galβ1-4GlcNAcβ1-3Gal

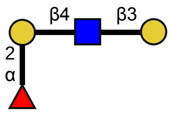

No binding detected

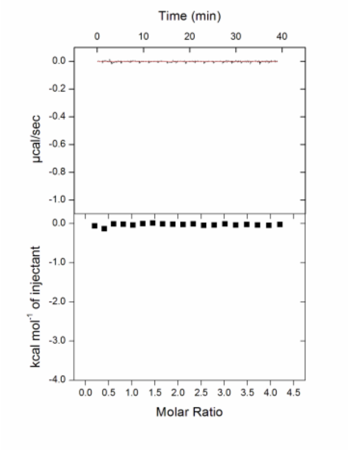

SLe^x antigen (pentasaccharide form)

Neu5Acα2-3Galβ1-4(Fucα1-3)GlcNAcβ1-3Gal

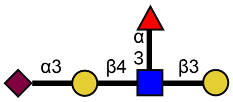

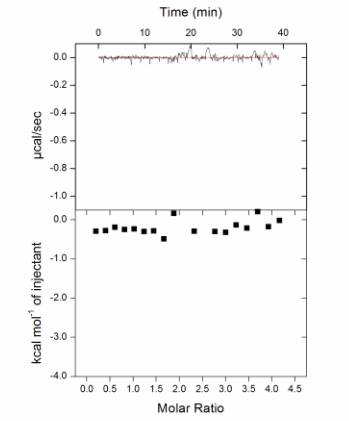

No binding detected

[a] Average of three independent experiments (SEM = ± 15µM).
[b] Average of two independent experiments (Range = ± 45µM).

Table S4. Oligonucleotides used in BabA cloning and site-directed mutagenesis. Where applicable, the recognition sites for the stated restriction enzymes are underlined. [FOR] and [REV] denote sense and antisense primers, respectively. [Phos] denotes a 5' phosphorylation.

**Designation**	**Sequence (5' – 3')**
BabA [FOR] 1- NcoI	TCGGATCCATGGAAGACGACGGCTTTTAC
BabA [REV] -527 BamHI	TCTGCTGGATCCCTTCTTCTTCTTCTTCTTGAGTTCTTG GTTGATGG
BabA D233A [FOR]	[Phos]GTTCAAAAGTGGTTGCTAGTCGTGCAGATG
BabA D233A [REV]	[Phos]TGATCGTGGTGGTTACGCTTTTGCCGTCTATG
BabA D233A/S244A [FOR]	[Phos]GTAATACAACAGGGGTGGCCTACACCGAAATCA C
BabA D233A/S244A [REV]	[Phos]CATCTGCACGACTAGCAACCACTTTTGAAC
BabA N206A [FOR]	[Phos]GAACCAAGACTAAAATCCAAACCATAGAC
BabA N206A [REV]	[Phos]CGTCTTTTTGAGCATTTACACCTGTGAC
